# Comparison of the whole slide imaging and conventional light microscopy in the grading of oral epithelial dysplasia: a multi-institutional study

**DOI:** 10.4317/medoral.23854

**Published:** 2020-11-28

**Authors:** Priscilla Barbosa Diniz, Marcondes Sena-Filho, Karen Mendes Graner, Bruno Augusto Linhares Almeida Mariz, Leonardo Amaral dos Reis, Oslei Paes de Almeida, Jacks Jorge

**Affiliations:** 1 Department of Oral Diagnosis, Piracicaba Dental School, University of Campinas (Unicamp), Piracicaba, SP, Brazil; 2 Botucatu Medical School, São Paulo State University (UNESP), Botucatu, Brazil

## Abstract

**Background:**

Whole Slide Imaging (WSI) is an alternative method to light microscopy (LM). However, few studies have compared the diagnostic agreement between WSI and LM, especially to grade oral epithelial dysplasia (OED). The purpose of this study was to evaluate the variability in grading OED by the World Health Organization grading system, using WSI and conventional LM, and to investigate whether the access to clinical information, and psychologic or physical states of the pathologists could interfere with the diagnosis.

**Material and Methods:**

eleven experienced pathologists from seven Brazilian universities independently evaluated twenty-five OED cases. The analyses were performed in duplicate for each method, with an interval of at least 30 days, and the time consumed in each analysis was measured. Physical and psychologic states were evaluated by blood pressure levels, heart rate and two questionnaires: State-Trait Anxiety Inventory and Perceived Stress Scale. Clinical information was provided after the second evaluation using WSI and the pathologist could change their diagnostic decision or not.

**Results:**

LM showed a higher inter-examiner agreement (k=0.53) than WSI (k=0.45) and a smaller time consumed by the pathologists (mean of 65.53 seconds compared to 91.02 seconds in WSI). In the first analysis using conventional microscopy, there was a positive correlation between kappa values and anxiety (r=0.47, *p*=0.02), and stress (r=0.64, *p*<0.01), and an inverse correlation with heart rate (r=-0.48, *p*=0.02). In the digital analysis, there was also a positive correlation between kappa values and anxiety (r=0.75, *p*<0.001). After clinical information was given, there was a slight change in 11.3% of the cases, and a great discrepancy in 1.1% of the cases, mainly increasing the OED grade.

**Conclusions:**

both microscopy systems had similar results, although LM had slightly higher kappa values, and WSI was more time consuming.

** Key words:**Pathology, microscopy, diagnosis, leukoplakia oral, anxiety.

## Introduction

The term oral potentially malignant disorder (OPMD) describes clinically detected epithelial lesions that carry an increased risk of progression to cancer (1-3). Oral epithelial dysplasia (OED) is one of the most common types the OPMD and its grading and diagnosis are basically made by the histopathological investigation, and lesions with a high grade of dysplasia are considered to be at higher risk of malignant transformation (3,4). Performing a correct diagnosis is the most important role for pathologists, and the literature reports three main causes for diagnostic mistakes: systemic causes, cognitive mistakes and lack of knowledge (5). The emotional state can have a negative cognitive impact at the moment of decision as well as the subjective aspect of the lesion, which is a reality in OED (6-8). In this context, access to clinical information, lesion site, and clinical images are imperative in the diagnostic process (9). The use of different methods of microscopic visualization can also influence lesion grading or even the final diagnosis (10,11).

Light microscopy (LM) is the classic method used by pathologists to achieve an accurate diagnosis. However, new methods have been developed in the last decades (12), helping pathologists to capture, visualize, analyze, store and share slides electronically, which are increasingly and easily available in most pathology centers (13-15). Whole Slide Imaging (WSI) has been used in Pathology for teaching and diagnostic purposes, including pathology conferences, consultations, reviews, slide panels, and more recently, for the initial diagnosis and archiving (14,16,17).

Considering all the advantages of WSI and the currently easier access to this technology, some studies have tried to validate its use in histopathology settings, but limited studies have compared the diagnostic agreement between WSI and traditional LM (14). Hence, our study aimed to evaluate the intra observer variability in grading OED by the World Health Organization (WHO) grading system, and the effects that pathologists’ physical and psychological states have in the diagnostic performance.

## Material and Methods

- Sample selection

Cases diagnosed between 2000 and 2015 as hyperkeratosis and acanthosis, epithelial dysplasia (mild, moderate or severe) or in situ carcinoma were selected from the Oral Pathology department of the Piracicaba Dental School, University of Campinas. Only cases with complete clinical data (age, sex, location, time of evolution, diagnostic hypothesis and clinical photograph) were selected by two pathologists that did not participate in the subsequent analysis. Inclusion criteria included: the diagnostic agreement between both pathologists, no technique errors, tissue integrity, and adequate coloration. Twenty-five cases, including 5 cases of hyperkeratosis and acanthosis, 16 cases of OED (6 mild, 6 moderate and 4 severe) and 4 cases of in situ carcinomas attending these criteria were selected and classified according to the WHO grading system.

- WSI imaging

Slides stained with hematoxylin and eosin (HE) were coded and scanned using the slide scanner Aperio ScanScope CS (Aperio Technologies, Vista, CA, USA). All images were checked for focus, brightness and contrast balance. All WSI analyses were performed using the software ImageScope version 11.2.0 Aperio®, in the same computer (Ultrabook Asus S46C, Intel® Core™ i5-33175 CPU @ 1.70GHz, 8 GB RAM). In the LM analysis, the pathologists used their usual microscope.

- Microscopic analysis

Thirteen pathologists were invited to participate in this study, and eleven voluntarily accepted. Six meetings were scheduled with each pathologist, two for methodological instructions and four meetings for actual study. Each slide was diagnosed twice using WSI or LM. All pathologists filled a form informing their age, time working as an oral pathologist, institution, and level of education (graduation, post-graduation, international experience). Before analyzing the study cases, ten control cases (including mucocele, fibrous hyperplasia, and squamous cells carcinoma) were selected to familiarize the pathologists with the research method. Initially, the study cases were analyzed by conventional LM by each pathologist. After 30 days, the pathologists analyzed the same cases digitally. After a minimum of 30 days interval, the second course of evaluations was performed to compare the intra observer agreement. The time spent for each analysis was measured. Pathologists and researchers were blinded to the slides and images identification. The slides were randomly identified by codes and the display order was changed for each course of the evaluation.

To assess the influence of clinical information on OED grading, clinical photos and clinical information were provided after the pathologists completed the second course of WSI. The slides were evaluated again and a final diagnosis was established for each case.

- Stress and anxiety analyses

Pathologists were evaluated for the presence and severity of stress and anxiety symptoms. A complete version of the Perceived Stress Scale (PSS), in the version translated to Portuguese by Luft *et al*., [2007] (18), was used to evaluate the symptoms of perceived stress in the month before the analysis. Anxiety was determined using the State-Trait Anxiety Inventory (STAI), a scale with 40 items, equally divided into two subscales (19). The first subscale evaluates the anxiety state (STAI-S) and expresses the feeling in the present moment. The second subscale is the trait (STAI-T) and evaluates sTable aspects of anxiety tendency, such as general states of calmness and confidence (20).

STAI-T was measured only in the first meeting with the pathologists. STAI-S and PSS were measured in all meetings before the analysis of the slides.

- Physical analysis

After the pathologists answered the questionnaires, blood pressure and heart rate were measured using a digital upper arm blood pressure monitor (Omron HEM-7113). This measurement was performed in all meetings, right before and right after the analysis of the slides. Normal values of arterial pressure and heart rate were considered normal (111/75 mmHg) and 73 bpm.

- Statistical analysis

Data were analyzed using the software SAS version 9.3 (SAS Institute, Cary, NC). Weighted Cohen´s Kappa was used to measure the intra-observer agreement, and it was interpreted as follows: Kappa < 0,10 (no agreement); 0,11 to 0,40 (weak); 0,41 to 0,60 (regular); 0,61 to 0,80 (moderate); 0,81 to 0,99 substantial (great) and Kappa =1,00 (perfect agreement). Multiple comparisons and the Tukey-Kramer test, Wilcoxon test, dependent T-test (for dependent data) and Pearson’s test were used in this study for association with the other variables. The statistical significance was set at *p* < 0.05.

## Results

- Participants characteristics

All participants were professors of Pathology in Brazilian Universities and were experienced in the histopathological routine. They were mostly males (54.4%), with a mean age of 46 years old (SD±10.77) and had an average of 19.8 years (SD±11.9) of working time. One pathologist had a medical degree and the other 10 were dentists; 27.3% of all participants were specialists in Pathology, almost all had MSc degrees (90.9%) and all had PhD. diplomas. Also, 54.5% had international experience and 63.6% had always worked in the same laboratory.

- Intraobserver Agreement

**Figure 1:**
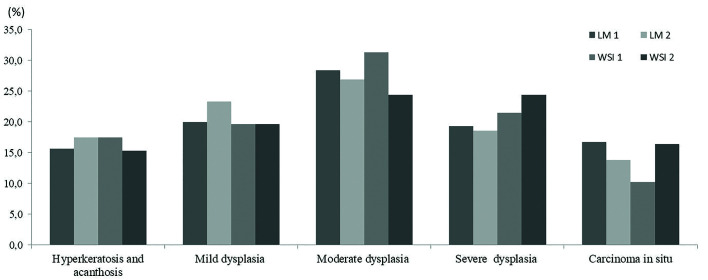
Frequency of diagnoses rendered using light microscopy and whole slide imaging.

The most frequent diagnosis of the study was moderate dysplasia (27.8%), followed by severe (20.9%) and mild dysplasia (20.6%), hyperkeratosis and acanthosis (16.5%), and carcinoma in situ (14.2%) (Fig. 1). Weighted Cohen´s Kappa showed a higher agreement rate in LM (k = 0.53, CI95% 0.46-0.60) than in WSI (k = 0.45, CI95% 0.38-0.51), representing a regular agreement for both methods. There was a variation of the diagnosis among pathologists by both methods, and this variation was statistically significant in both methods, but LM varied less (Chi-Square test *p* = 0.009) than WSI (Chi-Square test *p* = 0.0008).

- Time for diagnosis

The time for performing the diagnosis was evaluated for WSI and LM. OED; either mild, moderate or severe; consumed more time for diagnosis than other lesions. LM consumed on average 65.5 second for each case and was faster than WSI, which took a mean of 91.0 seconds (*p* <0.0001, Tukey-Kramer test). Moreover, the time for the diagnosis of each slide varied between the phases of the study. We found that in the first phase 84.5 seconds were spent in each case, while in the second phase the necessary time decreased to 72.1 seconds (*p*=0.002, Tukey-Kramer test).

- Effect of clinical information

To evaluate the importance of clinical information in the diagnosis, the pathologists first observed each slide without the clinical information and established their diagnosis. Then, this information was provided and the pathologists could change the diagnosis if necessary. Correlating the diagnosis in these two moments, there was a slight change in 11.3% of the cases, and a great discrepancy in 1.1% of the cases after receiving the clinical information. In most cases where the clinical information influenced the diagnosis, the pathologists tended to increase the grade of dysplasia, since the mean difference between the diagnosis was -0.101 (*p*<0.01, Wilcoxon test).

- Psychological characteristics

**Figure 2:**
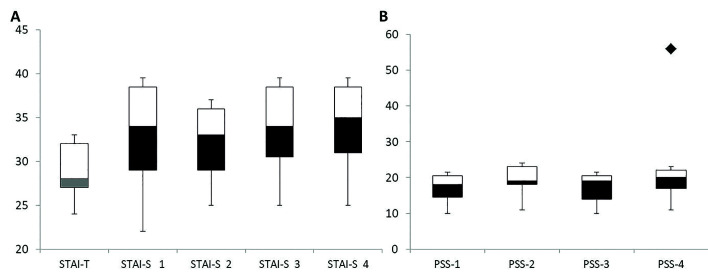
(A) Variation on T-Anxiety and S-Anxiety among pathologists in each stage of the study. S-anxiety 1 and 3 - Light Microscopy. S-Anxiety 2 and 4 - Whole Slide Imaging. (Scores: <33= mild anxiety; 33-49= moderate anxiety). (B) Variation of stress noticed among pathologists. One outlier was observed. Perceived Stress Scale (PSS) 1 and 3 - Light microscopy. PSS 2 and 4 - Whole Slide Imaging. Scores: 0-13= low stress; 14-28= mild stress; 29-42= moderate stress; 43-56= severe stress.

We observed that the pathologists were slightly anxious, as showed in the results of STAI-T, with a median score of 29.36 (SD±3.668) (20). When STAI-S was applied after each evaluation, an increase in the anxiety was observed, characterizing moderate anxiety (33.91, SD±6.5115) (Fig. 2).

The mean value of stress among the pathologists during this study was 19.47 (SD±6.65), characterizing mild stress. Stress levels were similar during all the study phases. There was only one outlier (Fig. 2).

When correlating the physical and psychological evaluations with the diagnosis agreement (kappa values) using Pearson's test, only in the first stage there was a positive correlation between kappa values and anxiety (r=0.47, *p*=0.02), and stress (r=0.64, *p*<0.01), and an inverse correlation with heart rate (r=-0.48, *p*=0.02). In the digital analysis, there was also a positive correlation between kappa values and anxiety (r=0.75, *p*<0.001).

## Discussion

The current study was conducted to analyze the variability on grading OED by the WHO grading system, using LM and WSI. Some studies compared WSI and LM using tissues from different anatomic sites, such as the gastrointestinal tract (11,12), breast (21), prostate (22), and skin (23). However, this is the first multi-institutional study comparing these two methods for the histopathologic grading of OED. Additionally, according to our results, LM still remains the main tool to evaluate and grade OED, presenting higher intraobserver agreement, and being less time consuming than WSI.

Cell morphology and tissue architecture are the main characteristics of grading OED in the diagnostic routine (24). Aspects such as nuclear atypia and mitotic Figures are used to determine the degree of dysplasia (25). The WSI method may result in difficulty identifying these features due to poor image resolution at high magnification, although we did not find that image quality was a major factor in the diagnostic discrepancy. Also, the lack of experience of pathologists using the WSI system is considered a factor for the discrepancies in the diagnoses between WSI and LM (26).

On the other hand, the variability in the diagnosis of OED was already expected due to the subjectivity of each pathologist’s analysis, regardless the use of different technologies (4,25). In our study, both WSI and LM methods showed similar results regarding the final diagnosis. The concordance among the pathologists in our study ranged from k=0.53 in LM to k=0.45 in WSI and was similar to previous studies using LM (24,25).

The WSI method was more time-consuming than LM in other studies (11,14,27), corroborating with our findings. This can be explained by lack of experience in the WSI method, and by the learning curve of new technologies (21). This limitation may discourage pathologists from adopting the digital platform in routine laboratory practice (21). We also observed that the first phase was more time consuming than the second, which corroborates the founds of Gui *et al*. (11), suggesting that the diagnosis of epithelial dysplasia using WSI may become more rapid with experience.

Generally, pathologists do not change their OED diagnosis even when they receive the patient's clinical information. There are few studies about the influence of clinical information in OED (9). Abbey *et al*. demonstrated that absence of clinical information did not interfere with the pathologists’ diagnosis of OED. The authors reported that the results could be more accurate, but it would not change their conclusions (9).

The diagnostic routine involves experience, skills, fatigue and competency, factors that can influence the decision of the pathologist (6). For decisions with more than one potential interpretation, more anxious people tend to behave more negatively than less anxious individuals with potential consequences for microscopic interpretation (7). We observed that in the first LM evaluation there was an increase in stress and anxiety after the diagnosis, which may have occurred due to tension or discomfort with a new and unknown situation (28). However, in the following evaluations, the pathologists became more confident with the methodology of the study. S-Anxiety was greater than T-Anxiety in all the stages of the study, which may be the result of an increase in attention levels (7).

WSI has been increasingly used for education, remote consultation, second opinions, and archiving (15,17,22). Despite the increased use of WSI in Europe and North America, there is a lack of available evidence to validate the use of WSI in routine primary diagnoses. The barriers to its implementation include cost-effectiveness, lack of evidence validating the diagnostic agreement and low acceptability among pathologists (27). Few institutions and laboratories have access and use this method in Brazil (29), although this method presents several advantages such as the off-site access to the digital slides, a quick and easy way to consult with other pathologists, to avoid lost or damaged slides, and enabling better ergonomics (30).

Some pathologists may find it more difficult than others to use WSI systems. We consider that our results show that pathologists can reliable grade OED using WSI, at similar levels to LM.

This is the first study to compare WSI with LM in grading OED. Our results indicate that both systems have similar results, although LM had slightly higher kappa values and was less time consuming. Conversely, more training and access to this new method could change these results. Further studies and more training are necessary to assess differences between WSI and LM in grading OED, and in other groups of oral lesions.
